# A neuromorphic implementation of multiple spike-timing synaptic plasticity rules for large-scale neural networks

**DOI:** 10.3389/fnins.2015.00180

**Published:** 2015-05-20

**Authors:** Runchun M. Wang, Tara J. Hamilton, Jonathan C. Tapson, André van Schaik

**Affiliations:** The MARCS Institute, University of Western SydneySydney, NSW, Australia

**Keywords:** mixed-signal implementation, synaptic plasticity, STDP, STDDP, analog VLSI, time-multiplexing, dynamic-assigning, neuromorphic engineering

## Abstract

We present a neuromorphic implementation of multiple synaptic plasticity learning rules, which include both Spike Timing Dependent Plasticity (STDP) and Spike Timing Dependent Delay Plasticity (STDDP). We present a fully digital implementation as well as a mixed-signal implementation, both of which use a novel dynamic-assignment time-multiplexing approach and support up to 2^26^ (64M) synaptic plasticity elements. Rather than implementing dedicated synapses for particular types of synaptic plasticity, we implemented a more generic synaptic plasticity adaptor array that is separate from the neurons in the neural network. Each adaptor performs synaptic plasticity according to the arrival times of the pre- and post-synaptic spikes assigned to it, and sends out a weighted or delayed pre-synaptic spike to the post-synaptic neuron in the neural network. This strategy provides great flexibility for building complex large-scale neural networks, as a neural network can be configured for multiple synaptic plasticity rules without changing its structure. We validate the proposed neuromorphic implementations with measurement results and illustrate that the circuits are capable of performing both STDP and STDDP. We argue that it is practical to scale the work presented here up to 2^36^ (64G) synaptic adaptors on a current high-end FPGA platform.

## Introduction

Plastic synapses, i.e., synapses that can adapt their gain according to one or more adaptation rules, are extremely important in neural systems, as it is generally accepted that learning in the brain arises from synaptic modifications. The Spike Timing Dependent Plasticity (STDP) algorithm (Gerstner et al., 1996; Magee, 1997; Markram et al., 1997; Bi and Poo, 1998), which is one of the adaptation rules observed in biology, modulates the weight of a synapse based on the relative timing between the pre-synaptic spike and the post-synaptic spike. Besides weight adaptation, some observations suggest that the propagation delays of neural spikes, as they are transmitted from one neuron to another, may be adaptive (Stanford, 1987). Axonal delays are an important feature that seems to play a key role in the formation of neuronal groups and memory (Izhikevich, 2006). In our previous work (Wang et al., 2011b, 2012), a delay adaptation algorithm, Spike Timing Dependent Delay Plasticity (STDDP), inspired by STDP was developed to fine-tune delays that had been programmed into the network. We recently showed that the time delays of neural spike propagation in the rat somatosensory cortex can be modified by suprathreshold synaptic processes such as STDP (Buskila et al., 2013). This suggests that it is likely that synaptic weights and the propagation delays are adapted simultaneously.

The main goal of this work is to develop a design framework that is capable of implementing neural networks with maximum size, using simplified biological models. To allow for future implementations that interface with the real world, these neural networks should be running in real time. While detailed simulations of small networks of neurons are one way of studying neural systems, such small networks are not able to capture all the complexity and dynamics of a large scale neural network with non-linear properties, such as a model of neocortex, as pointed out by Johansson and Lansner (2007). In the work reported here, we have therefore focussed on attaining maximum network size.

As synaptic plasticity has not yet been fully characterized and models of synaptic plasticity remain in flux (Brenner and Sejnowski, 2011; Sejnowski, 2012), dedicated hardware implementations that have been hardwired to one particular type of plasticity rule will not be able to adapt to likely future changes in plasticity models. Thus, the design framework we present here will be capable of including various substantial neural networks, each of which may be designed to solve a particular task.

In this paper, we will focus on exploring hardware friendly implementations rather than comparing our learning rules with the vast, well established complex algorithms used in computational neuroscience. As a result of our hardware focus, mathematical analysis of the long-term behavior of the plasticity rules in benchmark networks and quantifying the effects of our learning rules on the synaptic weight, which are commonly used in computational modeling papers, are out of the scope of this paper and will therefore not be addressed.

Simulating neural networks on computers has been successful in informing the computational neuroscience community on promising learning strategies, network configurations and neural models for many decades. This approach, however, does not scale very well, slowing down considerably for large networks with large numbers of variables. For instance, the Blue Gene rack, a two-million-dollar, 2048-processor supercomputer, takes 1 h and 20 min to simulate 1 s of neural activity in 8 million integrate-and-fire neurons (Izhikevich, 2003) connected by 4 billion static synapses (Wittie and Memelli, 2010). For smaller scale networks, Graphic Processing Units (GPUs) can perform certain types of simulations tens of times faster than a PC (Shi et al., 2015). GPUs still perform numeric simulations, however, and, depending on the complexity of the network, it can take hours to simulate 1 s of activity in a tiny piece of cortex (Izhikevich and Edelman, 2008). Along with general hardware solutions, there have been a number of more dedicated hardware solutions (Pfeil et al., 2012, 2013; Painkras et al., 2013). A good example of a dedicated solution that implements numeric simulation of neurons is the SpiNNaker project (Galluppi et al., 2012). In SpiNNaker, ARM processors run software neuron models. Their most recent work shows that the SpiNNaker cores are capable of implementing 96,000 synapses (7500 synapses per core) for STDP in real time (Galluppi et al., 2014).

An alternative approach is to use the analog VLSI (aVLSI) circuits, which avoid any need to discretise differential equations of neuronal dynamics. These implementations will also add stochasticity to the system through electronic noise and device mismatch, resulting in more realistic simulations of biological neural networks. The basic STDP learning rule, which is a paired pulse protocol (Gerstner et al., 1996), has been successfully implemented using aVLSI circuits (Bofill-i-petit and Murray, 2004; Indiveri et al., 2006; Häfliger, 2007; Koickal et al., 2007). More variants of the STDP algorithm have been proposed by Brader et al. (2007a) and Graupner and Brunel (2012). These algorithms capture more of the synaptic dynamics but still follow the principle that the modification of the synaptic weight depends on the relative timing of individual pre- and post-synaptic spikes. Many aVLSI implementations of these algorithms have been proposed (Chicca et al., 2003; Mitra et al., 2009; Giulioni et al., 2012). Similarly, aVLSI circuits have also been used to implement the STDDP learning rule (Wang et al., 2011a,b, 2012, 2013a). This aVLSI approach is useful for studying the dynamics of small and densely interconnected networks, but less so for the study of large and sparsely connected networks, such as complex models of various areas of cortex. The aVLSI implementations all used dedicated synapses for a specific type of synaptic plasticity and the number of plastic synapses integrated on single chip is usually fewer than tens of thousands. This significantly limits the size of the network these approaches can implement.

**Figure 1:**
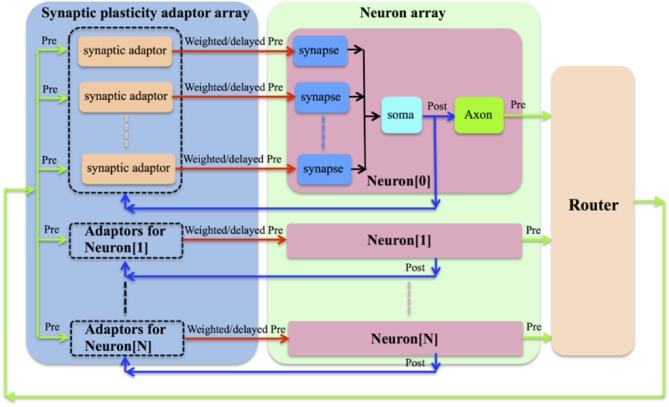
**The synaptic plasticity adaptor array that is separate from the neurons**. For each synapse, which remains part of the neuron, a synaptic adaptor will be connected to it when it needs to apply a certain synaptic plasticity rule. The synaptic adaptor will carry out the weight/delay adaptation by updating weight/delay values. For each incoming pre-synaptic spike, the adaptor will send a weighted/delayed pre-synaptic spike to the post-synaptic neuron in the neuron array.

We chose to implement a synaptic plasticity adaptor array that is separate from the neurons (see Figure 1). In this scheme, the address of the pre-synaptic spike from the pre-synaptic neuron will have already been remapped to the address of the post-synaptic neuron by the router shown in Figure 1. For each synapse, which remains part of the neuron, a synaptic adaptor will be connected to it when it needs to apply a certain synaptic plasticity rule. The synaptic adaptor will carry out the weight/delay adaptation by updating weight/delay values that are stored in digital memory. For each incoming pre-synaptic spike, the adaptor will send a weighted/delayed pre-synaptic spike to the post-synaptic neuron in the neuron array.

This strategy provides great flexibility, as a hardware neural network can be configured to perform multiple synaptic plasticity rules without needing to change its own structure, simply by connecting the synapses to the appropriate modules in the synaptic plasticity adaptor array. This structure was first proposed by Vogelstein and his colleagues in the IFAT project (Vogelstein et al., 2007). However, they didn't implement synaptic plasticity in that work although they did discuss the implementation of STDP with this structure. It seems that this flexibility will generate a communication overhead. The communication between neurons and adaptors has the same overhead as the communication between neurons and other neurons in a network without a separate adaptor array. Thus, the additional overhead stems from the communication from the adaptor array to each of the synapses. This will be discussed in more detail in the next section. The major disadvantage of our approach is that it is incapable of modeling the ion channels in the biological synapses. Compared to the aVLSI approach, our approach is less useful for studying the dynamics of the networks that require high degrees of biological realism. Furthermore, our approach is less power efficient compared to the aVLSI implementations. This is because our implementation has to employ configurable but power hungry devices, such as FPGAs/MCUs, to achieve its flexibility. Analog VLSI implementations, in contrast, especially those operating in weak inversion (Liu et al., 2002), are capable of achieving a significantly low power consumption.

We have previously presented a compact reconfigurable mixed-signal implementation of a synaptic plasticity adaptor that is capable of performing both STDP and STDDP (Wang et al., 2014a). Here, we present its follow-up work that uses a novel approach to scale up the numbers of synaptic plasticity adaptors up by 128 (2^7^) times more without increasing the hardware cost significantly. While the design of the router and the neuron arrays are out of the scope of this paper and will not be presented.

## Materials and methods

### Learning rules

#### Spike timing dependent plasticity

The STDP algorithm modulates the weight of a synapse based on the relative timing of the pre- and post-synaptic spikes. The weight of a synapse will be increased if a pre-synaptic spike arrives several milliseconds before the post-synaptic spike fires. Conversely, the weight will be decreased in the case that the post-synaptic spike fires earlier than the arrival of a pre-synaptic spike by several milliseconds. The amount and direction of modification of the weight are determined by the time between the arrival of the pre- and post-synaptic spike.

To obtain this time difference, we need to know when the pre- and post-synaptic spike arrives. This is implemented by introducing a time window generator, which is composed of a 4-bit counter, it will be reset by either spike and increased by one bit at each time step, e.g., 1 ms until it reaches its maximum value 0 × F. The time at which the alternative spike arrives is represented by the value of the counter. We also define that the time window is “active” before it reaches its maximum value. As we assume that the adaption will not be carried out if the pre- and post- synaptic spikes arrive simultaneously, only one time window generator will be needed.

In the original STDP learning rule (Gerstner et al., 1996), the amount of synaptic modification is summarized by the following equations:

(1) $$\Delta w=\;\{\begin{array}{c} A^{+}exp(\Delta t/\tau_{+}),\;if\;\Delta t<0\\ -A^{-}exp(\Delta t/\tau_{-}),\;if\;\Delta t\geq 0 \end{array}$$

where Δ*w* is the modification of the synaptic weight, Δ*t* is the time difference between the arrival time of the pre- and post-synaptic spike. The maximum amounts of synaptic modification Δ*w* are determined by two positive parameters: *A*^+^ and *A*^−^. The ranges of pre-to-post-synaptic interspike intervals over which synaptic modifications are performed are determined by the parameters τ_+_ and τ_−_. The authors in Song et al. (2000) concluded that this function provides a reasonable approximation of the dependence of synaptic modification on spike timing observed experimentally. However, it is a computationally intensive function since it requires exponentiation and division operations, both of which would occupy a large silicon area.

**Figure 2:**
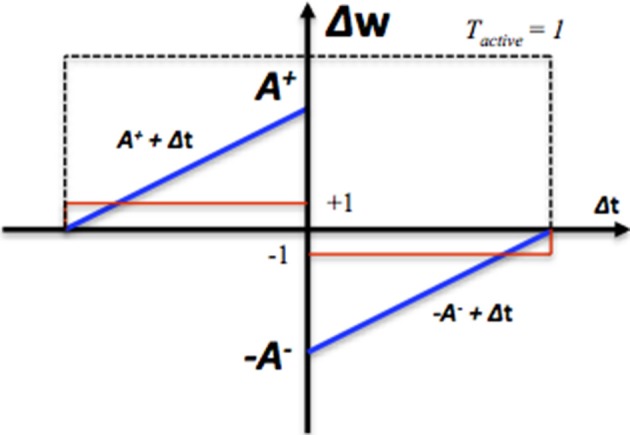
**The STDP modification function. Δ*t* is the time difference between the arrival time of the pre- and post-synaptic spike**. The blue line represents synaptic modification Δ*w*, which is linearly proportional Δ*t*. The red line represents the synaptic modification Δ*w*, which is a fixed step. The dashed line represents the range of pre-to-post-synaptic interspike intervals over which synaptic modification is performed.

To reduce the required silicon area, in our system, we have implemented two simplified modification rules. The first one is to change the weight proportionally to the calculated time difference (see the blue line in Figure 2) and is summarized by the following equations:

(2) $$\Delta w=\{\begin{array}{c} A^{+}\;+\;\Delta t,\;if\;T_{active}=1\;and\;\Delta t\;<\;0\\ -A^{-}\;+\;\Delta t,\;if\;T_{active}=1\;and\;\Delta t\;>\;0 \end{array}$$

where Δ*w* is the modification of the synaptic weight, Δ*t* is the time difference between the arrival time of the pre- and post-synaptic spike. *T_active_* is a Boolean value that indicates the time window generator is active (see the dashed line in Figure 2). In this system, the synaptic weight is an unsigned integer, which ranges from 0 to 15. *A*^+^ and *A*^−^ are both set to 16 here. The second one is to change the value of the weight by a fixed value (see the red line in Figure 2) and is summarized by the following equations:

(3) $$\Delta w=\{\begin{array}{c} +\;step,\;if\;T_{active}=1\text{ and }\Delta t<\text{0}\\ -\;step,\;if\;T_{active}=1\text{ and }\Delta t\;>0 \end{array}$$

where *step* is the fixed value and is set to 1 here. No weight modification will be performed if the pre- and post-synaptic spikes arrive simultaneously. The efficacy of these two simplified learning rules will be presented in Section Performance of STDP.

#### Spike timing dependent delay plasticity

**Figure 3:**
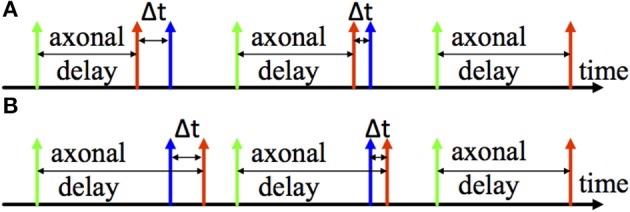
**Illustration of Delay adaptation. (A)** Delay increment; **(B)** Delay decrement. The axonal delay presents the delay between the firing time of the pre-synaptic neuron (the green one) and arrival time of the pre-synaptic spike (the red one) at the post-synaptic neuron. Δ*t* represents the time difference between the pre- and post-synaptic spike (the blue spike) to and from the post-synaptic neuron.

Two examples of the adaptation of axonal delays are shown in Figure 3, an increment of the delay (Figure 3A) and a decrement of the delay (Figure 3B). After the pre-synaptic neuron fires there is an axonal delay before the delayed pre-synaptic spike is sent to the post-synaptic neuron. If the post-synaptic spike, which is from the post-synaptic neuron, is not simultaneous with the delayed pre-synaptic spike, we may adapt the axonal delay by increasing or decreasing it by a small amount. This procedure is repeated until the delayed pre-synaptic spike occurs simultaneously with the post-synaptic spike.

Since this learning rule also needs to obtain the time difference between the pre- and post-synaptic spikes, we will use the same time window generator as described above, to generate the axonal delay. In this case, however, the time window generator will be started by the pre-synaptic spike (the green spike in Figure 3). Moreover, the duration of the generated time window will be modulated according to the axonal delay. The modification of the axonal delay will only be performed by the post-synaptic spike: when the post-synaptic spike arrives, if the time window is active, then there is a decrease the axonal delay and vice versa. The modification of the axonal delay Δ*d* is summarized by the following equations:

(4) $$\Delta d=\{\begin{array}{c} -\;step,\;if\;T_{active}=1\\ +\;step,\;if\;T_{active}=0 \end{array}$$

where *step* is a fixed value and is set to 1 here. Modifying the axonal delay by a single step is one of the three strategies, which were proposed and proved to be functional in our previous work (Wang et al., 2013b). No delay modification will be performed if the delayed pre-synaptic spike and post-synaptic arrive simultaneously. In this system, the axonal delay is also an unsigned integer, which ranges from 0 to 15.

### Design choice

To implement multiple synaptic plasticity rules for large scale spiking neural networks, the design choice we made were based on the following principles:

#### Time-multiplexing

In digital implementations of spiking neural networks, a single physical neuron can be time-multiplexed to simulate many virtual neurons, since digital hardware neurons can operate much faster than biological neurons. Each virtual neuron only needs to be updated every millisecond or so, as a millisecond time resolution is generally acceptable for neural simulations. Digital implementations of neurons using this time-multiplexing approach have been described in Cassidy and Andreou (2008); Cassidy et al. (2011); Wang et al. (2013b, 2014c). In the implementation presented here, we are time-multiplexing both the synaptic adaptors and the neurons.

#### Dynamic-assignment

It is not necessary to implement all neurons physically on silicon as based on the physiological metabolic cost of neural activity, it has been concluded that fewer than 1% of neurons are active in the brain at any moment (Lennie, 2003). A larger address space can be mapped onto a smaller number of physical components through dynamically assigning these components. Based on this principle, we have presented a dynamically-assigned digital and analog neuron array in Wang et al. (2013b) and Wang et al. (2014d), respectively. In these two systems, 4096 (4 k) neurons were achieved with only tens of neurons implemented physically on silicon. Here we also use this approach for both the neurons and the synaptic adaptors.

#### Mixed-signal

This implementation style can combine some of the advantages of both analog and digital implementations. Analog implementations can realize biological behaviors of neurons in a very efficient manner, whereas digital implementations can provide the re-configurability needed for rapid prototyping of spiking neural networks. As a result, mixed-signal implementations offer an attractive solution for implementing neural networks and many designs have been proposed for such systems (Goldberg et al., 2001; Gao and Hammerstrom, 2007; Mirhassani et al., 2007; Vogelstein et al., 2007; Harkin et al., 2008, 2009; Schemmel et al., 2008; Saighi et al., 2010; Yu and Cauwenberghs, 2010; Zaveri and Hammerstrom, 2011; Minkovich et al., 2012).

#### Standardization

To enable multiplexing building blocks, such as neurons, synapses, and axons, in a neuromorphic system, these circuits must be designed as standardized building blocks with a standard protocol for communication with programmable devices. Specifically for use in time-multiplexed neural systems, we have developed a synchronous Address Event Representation (AER) protocol, which uses a collision-free serial processing scheme with a single active signal and an address (Wang et al., 2013b). This synchronous scheme eliminates the overhead of an arbiter in the standard AER protocol.

For the maximum utilization of a fixed sized aVLSI chip, it is best to reduce the on-chip routing as much as possible as the routing can be carried out off-chip by FPGAs or microprocessors with more flexibility and extensibility. As the on-chip topology of the aVLSI circuits is generally fixed after fabrication, it is better to implement the whole system in an FPGA for prototyping and optimization before fabricating the aVLSI chips.

#### Pulse width modulation

For the systems that are sensitive to high communications overheads, e.g., aVLSI chips with limited number of pads, we adopted a pulse width modulation scheme, to minimize the communication bandwidth. In this scheme the durations of the spikes are modulated according to the synaptic weights, and the synapses in the neuron array are sensitive to the durations of the spikes (e.g., Wang et al., 2014b). It should be noted, however, that we could easily reconfigure the system to send out synaptic weights directly to the neurons in systems that are not sensitive to high communications overheads, e.g., FPGA designs.

#### Versatility

To efficiently implement synaptic plasticity in large-scale spiking neural networks with different learning rules, the building block should be capable of being configured for multiple synaptic plasticity rules, such as STDP and STDDP. When the synaptic plasticity adaptor is configured as the STDP adaptor, it performs STDP by receiving pre- and post-synaptic spikes from the pre- and post-synaptic neuron respectively. Its output, a weighted pre-synaptic spike generated using pulse width modulation, is sent to the synapse of the post-synaptic neuron for generating a post-synaptic current (PSC). When the synaptic plasticity adaptor is configured as an STDDP adaptor, it receives the same signals, but its output is a pre-synaptic spike that has been delayed according to the stored delay value for this neuron-to-neuron connection.

### Architecture

**Figure 4:**
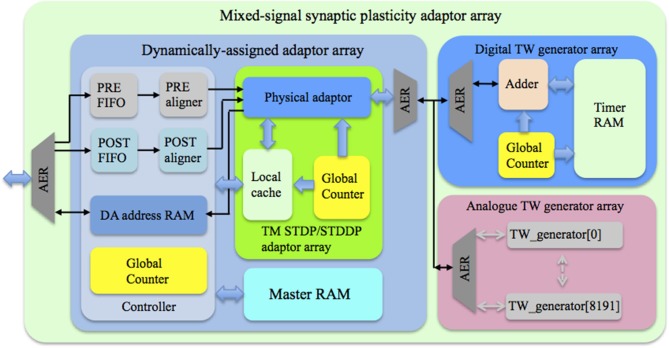
**Topology of the mixed-signal synaptic plasticity module array**. The controller receives pre- and post-synaptic spikes from the neuron array and assigns them to the corresponding TM adaptors according to their addresses. The global counter processes each TM adaptor sequentially. We use the Master RAM to store all the weight/delay values, while the TM STDP/STDDP adaptor array has a Local cache that stores the values of the DA adaptors that are being processed. The time window generator array generates the time windows that will be used by the TM adaptors for performing the learning rules.

Figure 4 shows the topology of the proposed mixed-signal synaptic plasticity adaptor array. It consists of an adaptor array on an FPGA and a time window generator array, which could be either a fully digital implementation on the same FPGA, or an analog implementation on a custom designed aVLSI chip, or both, as shown. All blocks use time multiplexing and are dynamically assigned using an FPGA to control the assignment.

Based on the physiological metabolic cost of neural activity, it has been concluded that fewer than 1% of neurons are active in the brain at any moment (Lennie, 2003). The anatomical studies of neocortex presented in Scannell et al. (1995) showed that cortical neurons are not randomly wired together. Instead, cortical neurons are typically organized into local clusters called minicolumns, which are then grouped into modules called hypercolumns (Hubel and Wiesel, 1974; Amirikian and Georgopoulos, 2003). The connections of the minicolumns are highly localized so that connectivity between two nearby (less than 25–50 μm apart) pyramidal neurons is high and the connectivity between two neurons drops sharply with distance (Holmgren et al., 2003). Based on the experimental data in Tsunoda et al. (2001) and Johansson and Lansner (2007) concluded that *at most a few percent of the hypercolumns and hence only about 0.01% of the minicolumns and neurons are active in a functional sense (integrating and firing) at any moment in the cortex*. They also concluded that only 0.01% of the synapses in our brains are active (transmitting signals) on average at any moment. Hence, in principle, one hardware synapse could be dynamically reassigned to 10^4^ virtual synapses on average. Such a hardware synapse will be referred to as a physical synapse and the synapse to be simulated will be referred to as a dynamically-assigned (DA) synapse. If a DA synapse cannot be simulated in a single time step, the physical synapse needs to be assigned to that DA synapse for a longer time and the number of DA synapses a single physical synapse can simulate will go down proportionally.

On an FPGA running at 200 MHz, we can time-multiplex a single physical synapse to simulate 1 ms/5 ns = 200,000 time-multiplexed (TM) synapses, each one updated every millisecond. Therefore, theoretically, a TM synapse array with 200,000 TM synapses can be dynamically assigned for 200,000 × 10^4^ = 2 × 10^9^ DA synapses, if these synapses can be simulated in a single 5 ns clock cycle and if only 0.01% of the synapses are active at any time step.

Since we chose to implement a synaptic plasticity adaptor array that is separate from the neurons, we will apply these two approaches to the adaptors. To be able to deal with higher synaptic activity rates, and because powers of two are preferable to optimize memory use for storing variables, such as weights and delays, we chose to dynamically assign one TM adaptor for 8192 (8 k) DA adaptors. The maximum active rate of the synapses that this system can support is therefore 1/8 k ≈ 0.012%. The TM adaptor array itself is configured to simulate 8 k TM adaptors, allowing it to support 8 k × 8 k = 64 M synapses. Each TM adaptor can use up to 25 clock cycles to complete its processing to maintain an update rate of 1 kHz (the corresponding time step is about 1 ms). The time window generator array is also configured to have 8 k identical time window generators, each time window being assigned to one TM adaptor.

The dynamically-assigned adaptor array consists of three sub-blocks: a controller, a TM STDP/STDDP adaptor array and a Master RAM. A single physically implemented dynamically-assigned adaptor array is capable of representing up to 64M DA adaptors, thus the hardware cost of the DA adaptors is negligible. The physical constraint for this approach is data storage. On-chip SRAM (on an FPGA) will be highly limited in size (generally less than tens of MBs), while the use of off-chip memory will be limited by the communications bandwidth. It is difficult, but not impossible, to use off-chip memory with the time-multiplexing approach, as new values need to be available from memory every time slot to provide real-time simulation.

Since we are aiming for the maximum network size, we need to ensure that the system is able to utilize off-chip memory. Inspired by the cache structure used in state-of-the-art CPUs, we use the Master RAM to store all the weight/delay values, while the TM adaptor array has a Local cache that stores the values of the DA adaptors that are being processed. The accessing (read/write) of the Master RAM will only be performed when needed. This means that new values are no longer required to be available from memory every time slot. Hence this cache structure greatly reduces the bandwidth requirement to use external memory. We will present the details of this cache structure following a presentation on the management the incoming spikes. It should be noted, however, that using off-chip memory requires flow control for the memory interface, which results in a more complex system architecture. Thus, for the work reported here, we use only on-chip memory, thus simplifying the system architecture. We will discuss the usage of off-chip memory in more detail in Section Discussion.

The controller receives pre- and post-synaptic spikes from the neuron array (see Figure 4) and assigns them to the corresponding TM adaptors according to their addresses. In our previous work (Wang et al., 2013b, 2014d) that also implemented the dynamic-assignment algorithm, the controller needs to check whether there is already a neuron assigned to the incoming spike or not. This method has a high usage of slice LUTs, which is the bottleneck for large-scale FPGA designs. This is because that method requires an address register array and a timer array, both of which are running in parallel and hence have to be implemented with slice LUTs.

To avoid this problem, we chose instead to use a direct mapping method that assigns one fixed TM adaptor as the target adaptor for the incoming spike irrespective of whether the TM adaptor has been assigned or not. The incoming spike's AER address is a 26-bit address (along with a single active line). We only store the most significant 13 bits out of 26 bits into a DA address RAM (a dual port RAM with a size of 8 k × 13 bits), while the other 13 bits determine where, i.e., in which position of the DA address RAM, the 13 bits will be stored.

To decouple writing new events (pre- and post-synaptic spikes) from reading out from current events, we use a FIFO and an aligner (a dual port RAM with a size of 8 k × 1 bit that corresponds to 8 k TM adaptors). For pre- and post-synaptic spikes, the size of the FIFO is 16 × 26 bit and 16 × 13 bit respectively. The work presented in Cassidy et al. (2011) used two banks of dual port RAM to implement a ping-pong buffer. This requires much more RAM than our solution.

**Figure 5:**
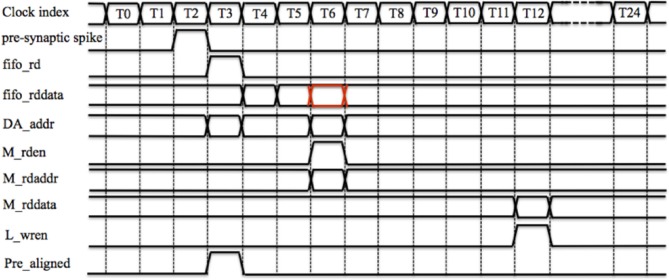
**The controller's timing diagram of one time slot**. A pre-synaptic spike arrives at the controller at T2 and it will be written into the PRE_FIFO. The controller will read the PRE_FIFO at T3 and the read data (fifo_rddata) will be ready at T4. To indicate that a spike has arrived (for that TM adaptor), at T4, controller will write 0 × 1 into the PRE_aligner to the position determined by the least significant 13 bits of the fifo_rddata. The controller will also use the least significant 13 bits of fifo_rddata to retrieve the stored address (from the DA address RAM), which will be ready at T6. At T6, the controller will read the Master RAM by asserting a read enable signal (M_rden) with a read address M_rdaddr, which is the fifo_rddata signal delayed (the red one). At T12, the output from the Master RAM M_rddata will be ready and the Local cache will be updated by asserting L_wren.

Figure 5 shows the timing diagram for the controller for one time slot. Assuming the PRE_FIFO is empty at T0, when a new pre-synaptic spike arrives (its active line is high) at T2, its 26-bit AER address will be written into PRE_FIFO. The controller will then read the PRE_FIFO by asserting fifo_rd at T3 (since the PRE_FIFO is not empty anymore) and read data (fifo_rddata) will be ready at T4 (one clock cycle latency). To indicate that a spike has arrived (for that TM adaptor), at T4, the controller will write 0 × 1 into the PRE_aligner to the position determined by the least significant 13 bits of the fifo_rddata.

At T4, the controller will also use the least significant 13 bits of fifo_rddata to retrieve the stored address (from the DA address RAM), which will be ready at T6 (two clock cycles latency). If this retrieved address does not match the most significant 13 bits of the delayed fifo_rddata (the red one), this indicates that the target DA adaptor is not the one that has been assigned before. Hence the value (in the Local cache) of the TM adaptor needs to be updated with the value of the target DA adaptor. Therefore, at T6, the controller will read the Master RAM by asserting a read enable signal (M_rden) with a read address M_rdaddr, which is the fifo_rddata signal delayed. For the same reason, at T6, the controller will also update the DA address RAM with the address of this newly arrived pre-synaptic spike: the most significant 13 bits of the delayed fifo_rddata (the least significant 13 bits determines the position to write). The output from the Master RAM M_rddata will be ready 6 clock cycles later (we will explain why this latency is needed Section TM STDP Adaptor Array) and the Local cache will be updated by asserting L_wren.

To read out the aligned pre-synaptic spike (Pre_aligned), at each time slot, the controller will read out the TM adaptor of that time slot, from the PRE_aligner at T1. The Pre_aligned will be ready at T3 where the corresponding TM adaptor will acknowledge it, after which it will be cleared. To avoid the collision that can happen when the PRE_FIFO and the controller both try to write the PRE_aligner (at the same location, although this case will not happen frequently) at T3, we set it so that the PRE_FIFO cannot be read (fifo_rd cannot be asserted) at T0. This is indeed the reason to introduce the FIFO since in this way all operations are fully pipelined and can be performed on every clock. To keep consistent with this pipeline, in each time slot, the controller will read out the DA_addr (no clear operation needed) for the TM adaptor of that time slot from the DA address RAM at T1. When the TM adaptor generates a weighted/delayed pre-synaptic spike, the controller will send this spike to the post-synaptic neuron with a 26-bit AER address, which is a combination of the DA_addr and the value of the global counter.

The timing for post-synaptic spike is aligned using a very similar scheme to that described above for the pre-synaptic spike, however, its address will not be stored in the DA address RAM. This is due to the fact that only the weighted/delayed pre-synaptic spike will be sent to the post-synaptic neuron (see Figure 1) and hence only the address of the pre-synaptic spike needs be stored and only the pre-synaptic spike will retrieve the weight/delay value from the Master RAM.

This method significantly reduces the usage of the Slice LUTs and hence makes it practical to apply the dynamic-assignment approach to an adaptor array with 8 k neurons. The hardware cost of the FIFO and the aligner is very small and they are both efficiently implemented with on-chip distributed SRAM. It does need a DA address RAM, which needs to be implemented with on-chip block SRAM, but storing only 13 bits significantly reduces the size of the address memory. Another major advantage of this method is its flexibility, e.g., with a 26-bit AER address, input spikes can arrive at any time and be handled. This means multiple different types of neuron arrays can be connected to one adaptor array. Moreover, it suffers little from the large latencies in the spikes, that can be of the order of hundreds microseconds, due to routing. Excessive latency due to routing is quite common in large-scale neural networks. Similarly, the communication overhead between the neuron array and the adaptor array will barely affect the performance of the system.

A collision will happen when multiple input spikes, that target different DA adaptors, while at the same time need the same TM adaptor, arrive within one time step. In this case, only the last arriving spike will be sent to its target adaptor, and the ones that arrived previously will be simply discarded. Another collision will happen when the most significant 13 bits of the address of an incoming post-synaptic spike do not match the DA_addr of the target TM adaptor. In this case the 13 bits of the address of the post-synaptic spike will still be sent to that TM adaptor for performing adaptation and might cause wrong weight/delay modifications. These two possible collision scenarios are drawbacks of our approach, and do affect small and densely interconnected neural networks with high activity rates. These scenarios, however, are not serious problems for large-scale neural networks, the connections of which are highly localized, while the activity rate is low. For instance if we are modeling hypercolumns in human cortex, the experimental data shows that only a few hypercolumns in the human cortex are active for any given task.

For practical applications, within a short period, the TM adaptors should only be assigned for one certain task. When that task ends, they will be released and can then be used by other tasks. For example, one hypercolumn could use all the 8 K TM plastic synapses for learning patterns, which might last for hundreds of milliseconds. After the patterns have been learned (stored in the Master RAM), another hypercolumn could then use these 8 K TM adaptors for learning patterns. It is of course possible that a synapse in another hypercolumn becomes active more or less spontaneously. These spontaneously activated synapses, however, would be uniformly distributed all over the neural network and are thus unlikely to make up a large fraction of the group of synapses in the hyper column that is currently learning patterns. Hence, these spontaneously activated synapses will not have a significant effect on the learning being performed. We will validate the dynamic-assignment scheme in Section Validation of the Dynamic-assignment Scheme. The maximum memory update speed, which is indeed the maximum firing rate of the neurons, that our system supports is 200 Mhz/25 = 8 MHz (much higher than biological neurons).

### Time window generator array

The time window generator array has been successfully implemented on a custom designed aVLSI chip, and independently also on the same FPGA as the dynamically-assigned adaptor array. The digital implementation used time-multiplexing to achieve 8 k TM time window generators. However, this fully digital implementation needs block SRAM, as the internal state of each generator needs to be stored in memory in between updates. This memory demand is the real bottleneck of the time-multiplexing approach (Moore et al., 2012). Nevertheless, this fully digital solution will be quite suitable for the applications when aVLSI is not available. On the other hand, an aVLSI circuit can implement a time window generator very efficiently, as long as high precision is not required. Using the aVLSI time window generator circuit reduces memory usage and the memory saved can be used for storing more synaptic weight and delay values, allowing for larger networks. Furthermore, the analog time window generator will add stochasticity to the weight and delay adaptation through electronic noise and device mismatch, which will provide more realistic simulations of biological neural networks.

#### Analog time window generator array

**Figure 6:**
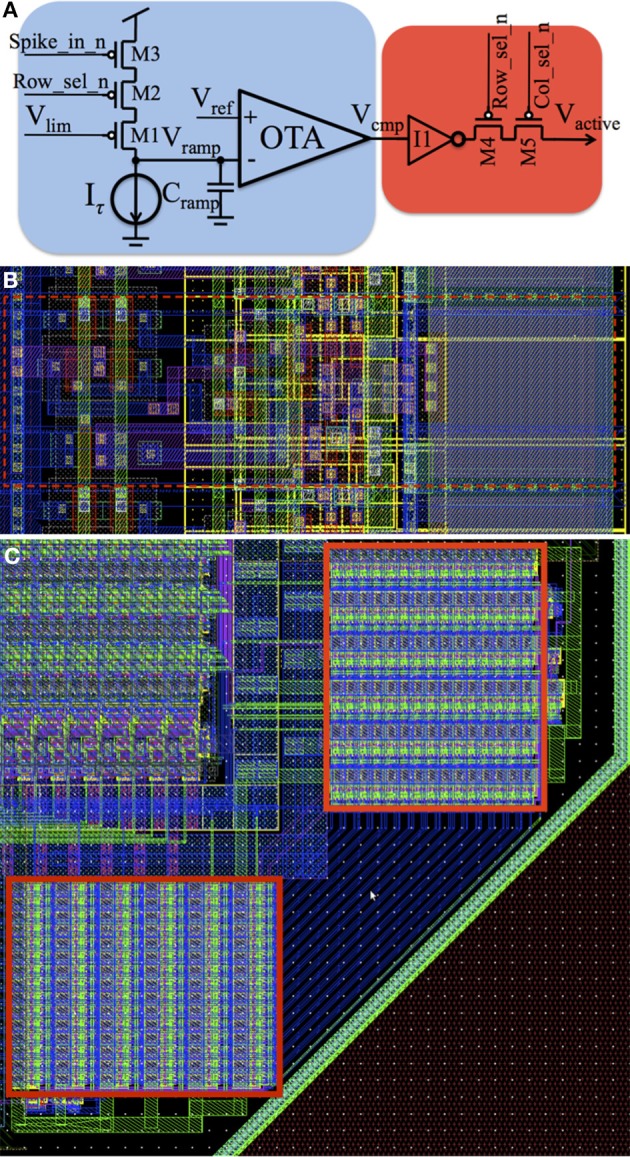
**aVLSI time window generator**. **(A)** Schematic; **(B)** Layout; **(C)** Layout of the array. It is placed in a two-dimensional array and when a time window generator is selected, the voltage at node *V_cmp_* will pull the output signal of this neuron *V_active_* either up to *V_dd_* (active, *V_cmp_* is low) or down to ground (inactive, *V_cmp_* is high) via an inverter (I1) and a serial switch (M4–M5).

We provide a brief review of the analog time window generator, which has been presented in depth in Wang et al. (2014a). Figure 6A shows the schematic of the analog time window generator, comprising a ramp generator circuit (blue) and an AER hand-shaking circuit for our synchronous AER (red). It is placed in a two-dimensional array and therefore requires row and column select signals (*Row_sel_n* and *Col_sel_n*), which are both low when the ramp generator has been selected. When a time window generator is selected, the voltage at node *V_cmp_* will pull the output signal of this neuron *V_active_* either up to *V_dd_* (active, *V_cmp_* is low) or down to ground (inactive, *V_cmp_* is high) via an inverter (I1) and a serial switch (M4–M5). When this time window generator is not selected (M4 and M5 are OFF), *V_active_* will be driven by another other time window generator in the array. Each time window generator is linked to its corresponding TM adaptor and will be processed sequentially, with each generator selected for one time slot. To use the asynchronous aVLSI circuits with the FPGA, synchronization with its clock domain is needed. Since the output signal *V_active_* is a 1-bit signal, we use the general method that uses two serially connected flip-flops to sample the input (Weste and Harris, 2005).

This circuit was implemented in the IBM 130 nm technology. For the maximum utilization of silicon area, one time window generator should share as many resources as possible with its neighboring ones. Based on this principle, all the pMOS transistors are located in the right side and all the nMOS transistors are located at the left side (see the dashed red rectangle in Figure 6B) so that they can share their bulk connections with each other. All the input/output signals and the bias currents are placed vertically so that they can be merged to a bus across the array without any extra wiring cost. The effective size of a time window generator in the array is ~50 μm^2^ achieving a density of 20,000 cells/mm^2^. As a proof of concept, we have placed 180 of the proposed aVLSI time window generators on the bottom right corner of a test chip, as shown by the red rectangles in Figure 6C (Wang et al., 2014b).

#### Digital time window generator array

The digital time window generator has the exact same function as the aVLSI time window generator. The global counter processes each TM time window generator sequentially. In each time slot, the controller will read the value of the TM time window generator from the Timer RAM. A counter will be incremented by one at each clock cycle when the digital input spike from the time-multiplexed adaptor is active (high), so that its count increases proportional to the spike width. When there is no input spike, the count will decrease by one each time slot, until it reaches zero, indicating the end of the time window.

Slightly different to the aVLSI time window generator, the output of the digital time window generator contains not only an active line, to indicate whether the time window is finished or not, but also the actual value of the counter. In aVLSI it would be difficult to read out the actual value of the *V_ramp_* (see Figure 6A) in an efficient manner. In a digital implementation this value is directly accessible to the DA adaptor array and could be used to perform more complex plasticity rules.

### TM adaptor array

#### TM STDP adaptor array

When implementing the TM adaptor array for STDP, a significant reduction in memory usage was achieved by storing a bistable weight in the Master RAM. This is based on the work by Brader et al. (2007b), which shows that from a theoretical perspective, having only two stable states for synaptic weights does not degrade the performance of associative networks, if the transitions between the stable states are stochastic. For networks with large numbers of neurons, each with large numbers of synapses, the assumptions that synaptic weights will be discretized to two stable values on long time-scales is not too severe, and is supported by biological evidence (Bliss and Collingridge, 1993; Petersen et al., 1998).

**Figure 7:**
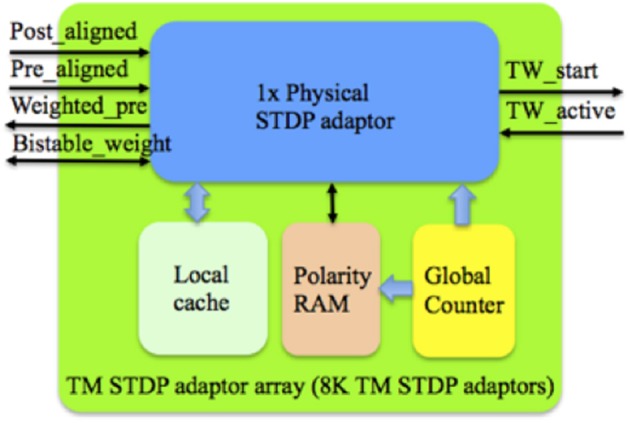
**Structure of the TM STDP adaptor array**. The global counter processes each TM STDP adaptor sequentially. The Local cache stores the weight values of the TM STDP adaptor that are being processed. The learning rules will be performed with the aligned pre- and post- synaptic spikes from the controller and the active line from the time window generator.

Figure 7 shows the structure of the TM STDP adaptor array, which consists of a physical STDP adaptor, a Local cache, a polarity RAM and a global counter. The Local cache, which is a dual port RAM with a size of 8 k × 4 bit, stores the weights (4-bit resolution) of the TM adaptors. The Master RAM will only need to store one of the two stable values of the bistable weight, and thus needs only 1 bit per weight. When a TM adaptor has been assigned to a DA adaptor, its bistable weight will be read out from the Master RAM. We then use this bistable weight to generate one random 4-bit weight (stored in the Local cache) for that TM adaptor. Only when there is a modification of the 4-bit weight, which will generate a bistable weight simultaneously, will we need to update the Master RAM with this bistable weight.

Since there is only one time window generator per TM adaptor, it will have the wrong weight modifications when multiple spikes (of the same type, e.g., pre-synaptic spikes) arrive within the duration of one time window. For instance, one pre-synaptic spike starts a time window while the pre-synaptic spikes that follow and arrive within this time window will perform weight decrement. To solve this problem, the polarity RAM, which is a dual port RAM with a size of 8 k × 1 bit, was introduced. The polarity RAM stores the polarity of the time window for each TM adaptor. For the time window started by pre- and post-synaptic spikes, the polarity value is 0 × 0 and 0 × 1 respectively. The time window is set such that it will be restarted for each of the multiple spikes received within the time window. In other words, each incoming spike will either start a time window or perform weight modification.

The read out from the Master RAM and the update of the Local cache was presented with the timing diagram of the controller in Section Architecture. Since the retrieved bistable weight from the Master RAM is 1-bit while the weight to be written into the Local cache is 4-bits, this bistable weight will be used as the most significant bit (MSB) of that 4-bit weight. To keep the transitions between the stable states stochastic, the remaining 3 bits are generated pseudo-randomly by a linear feedback shift register (LFSR).

**Figure 8:**
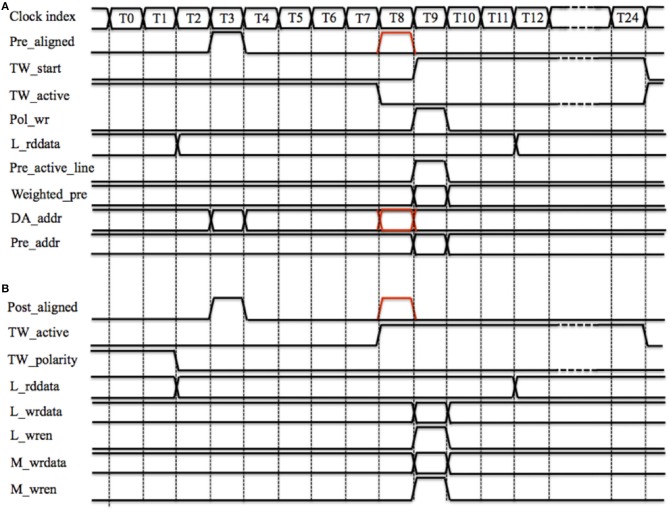
**TM STDP adaptor's timing diagram of one time slot. (A)** Starting a time window. Pre_aligned is ready at T3 and TW_active will be ready at T8. As the time window is inactive, the delayed Pre_aligned (the red one) will start the time generator at T8 by sending a pulse (TW_start, starts at T9); **(B)** Increasing weight. The polarity of the time window (TW_polarity) and the local weight (L_rddata) are both read out at T0 and ready at T2. Post_aligned is ready at T3 and TW_active will be ready at T8. The delayed Post_aligned (the red one) will increase L_rddata.

Figure 8 shows the timing diagram for performing the STDP algorithm by one TM STDP adaptor. Figure 8A shows how a pre-synaptic spike starts a time window. Pre_aligned is ready at T3 and TW_active will be ready at T8 (comprising 7 clock cycles for latency and 2 clock cycles for synchronization). As the time window is inactive, the delayed Pre_aligned (the red one) will start the time generator at T8 by sending a pulse (TW_start, starts at T9) that controls the duration of the window. Note the duration of the time window is fixed during operation but the parameter is configurable. The polarity of the time window, which is 0 × 0, will be written to the polarity RAM by asserting Pol_wr at T9. Since the time window is inactive, no weight modification is needed and neither the Local cache nor the Master RAM needs to be updated.

Since the incoming spike is a pre-synaptic spike, we need to generate the weighted pre-synaptic spike, which will be sent to the post-synaptic neuron. The local weight (L_rddata) is read out at T0 and ready at T2 (two clock cycles latency), the delayed Pre_aligned will send out the Weighted_pre and assert its active line (Pre_active_line) at T9. Simultaneously, the controller will send this spike to the post-synaptic neuron with a 26-bit AER address (Pre_addr), which is a combination of the delayed DA_addr (the red one) and the value of the global counter.

Figure 8B shows the timing diagram for increasing the synaptic weight. Assuming the time window has already been started by a previous pre-synaptic spike. The polarity of the time window (TW_polarity) and the local weight (L_rddata) are both read out at T0 and ready at T2 (two clock cycles latency). Post_aligned is ready at T3 and TW_active will be ready at T8. Since TW_active is active and TW_polarity is low, which indicates that this time window was started by a pre-synaptic spike, the delayed Post_aligned (the red one) will increase L_rddata by one (when using the aVLSI time window generator array) or by the value of the time window generator's counter (when using the digital time window generator array).

The updated weight (L_wrdata) will be written into the Local cache by asserting L_wren at T9. In the controller, the latency from fifo_rd, which cannot be asserted at T0, to L_wren is 10 clock cycles (see Figure 5). Hence a collision when the TM STDP adaptor and the controller are updating the Local cache at the same cycle will never happen. This is why the latency from M_rden to M_rddata is set to 6 clock cycles. The idea behind this setting is to achieve a fully pipelined design so that all operations can be performed on every clock cycle and there are no stalls in the pipeline. Note that if we were using only a digital time window generator array, the time slot could be optimized to less clock cycles by using tens of pipeline stages (Wang et al., 2013b, 2014c); it is the serial scanning of the aVLSI time window generator array that needs 25 cycles, as for any given time window generator, it has to be selected during the whole time slot. To maintain an architecture that is compatible with both the aVLSI and the digital time window generator array, we chose to use the time slot with 25 cycles for the work reported here.

Also at T9, the bistable weight will be updated to 1 if the weight is larger than a threshold, a pseudo random 4-bit number between 4 and 11 that is updated every time slot. Otherwise, the bistable weight will be updated to 0. The updated bistable weight will be written into the Master RAM by asserting M_wren at T9.

#### TM STDDP adaptor array

**Figure 9:**
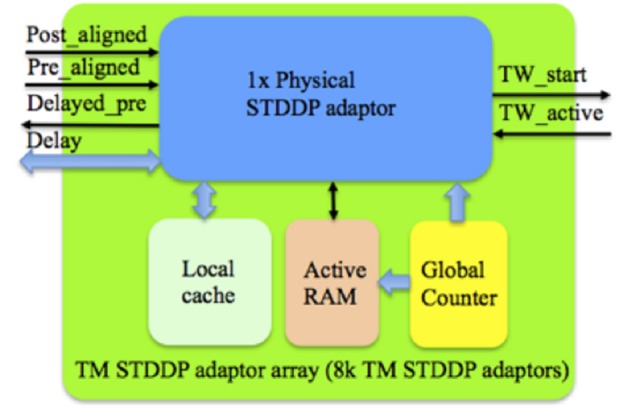
**Structure of the TM STDDP adaptor array**. The global counter processes each TM STDDP adaptor sequentially. The Local cache stores the axonal delay values of the TM STDDP adaptor that are being processed. The learning rules will be performed with the aligned pre- and post- synaptic spikes from the controller and the active line from the time window generator.

The TM STDDP adaptor array operates in the same scheme (with the same pipeline stages) as the TM STDP adaptor array. From the controller's point of view, they are identical. This means that they are interchangeable, which was a deliberate design decision. Figure 9 shows the structure of the TM STDDP adaptor array, which consists of a physical STDDP adaptor, a Local cache, an active RAM and a global counter. The Local cache, which is a dual port RAM with a size of 8 k × 4 bit, stores the 4-bit delay values of TM adaptors. The Master RAM stores the 4-bit delay values too. When a TM adaptor has been assigned to a DA adaptor, the delay of the latter will be read out from the Master RAM and then stored in the Local cache as the delay of that TM adaptor. When there is a modification of the delay the Master RAM is updated with the new delay.

Since the TM STDDP adaptor array pipeline is the same as the one presented for STDP earlier, the timing diagram is exactly the same as the ones presented in Figure 8 (replacing “weight” with “delay”) with the following additional changes:

1. Only the pre-synaptic spike can start the time window generator by sending it a spike with a duration proportional to the retrieved axonal delay.
2. The delayed pre-synaptic spike should be generated at the falling edge of the active line (from 0 × 1 to 0 × 0), which indicates the end of the axonal delay. Since this is a time multiplexing system, each TM adaptor will only know the value of the active line in the current time slot. To solve this problem, we introduced the active RAM, which is a dual port RAM with a size of 8 k × 1 bit, to store the value of the active line in the current time slot. While the retrieved value from the active RAM represents the previous value. The delayed pre-synaptic spike will be generated if the active line is low and the active line retrieved from the active RAM is high. For this reason, the actual axonal delay will be from 1 to 16 ms while the value of the delay stored is from 0 × 0 to 0 × F. The signals of the polarity RAM (see Figure 8) are replaced by those of the active RAM. The weight of this spike will be a fixed but configurable value.
3. Only the post-synaptic spike can change the delay. No adaptation will be performed if the falling edge of the active line has been detected at T8 since this means the delay has been perfectly tuned and a delayed pre-synaptic spike will be generated at T9.

### Utilization

The digital parts of the proposed array were developed using the standard ASIC design flow and therefore can be easily implemented with state-of-the-art manufacturing technologies. A bottom-up design flow was adopted in which we designed and verified each module separately. Once the module level verification was complete, all the modules were integrated together for chip-level verification. As a proof of concept, we implemented the proposed system on a Virtex6 XC6VLX240T FPGA, which is hosted on the Xilinx ML605 board. Table 1 shows the utilization of hardware resources on the FPGA. Note that this is the utilization for the dynamically-assigned STDP/STDDP adaptor array (without the Master RAM), the digital time window generator array, and the interface circuit for the aVLSI time window generator. As Table 1 shows, the proposed system uses only a small fraction (<1%) of the hardware resources. Limited by the size of the on-chip SRAM, for STDP and STDDP, we have implemented 1800 × 8 k = 14.4 M and 450 × 8 k = 3.6 M DA adaptors respectively. This is a proof of concept and in the future we will implement the Master RAM with off-chip memory, thus leveraging the design of the cache structure introduced.

**Table 1:** **Device utilization Xilinx Virtex6 XC6VLX240T**.

**Resource**	**STDP**	**STDDP**	**Total available**
Occupied slices	558(1.4%)	545(1.4%)	37,680
Slice FF's	398(0.1%)	399(0.1%)	301,440
Slice LUTs	1430(0.9%)	1422(0.9%)	152,720
LUTs as logic	578(0.3%)	568(0.3%)	152,720
LUTs as RAM	827(1.4%)	827(1.4%)	58,400
36 k RAM	5(1.2%)	5(1.2%)	416

## Results

For testing purposes, a PCB was developed as a daughter board to contain the aVLSI chip and was connected to the FPGA. The FPGA is controlled by a PC via a JTAG interface and the analog bias inputs of the aVLSI chip are controlled by external programmable bias voltages.

### Performance of STDP

We have tested the performance of the dynamically-assigned STDP adaptor array by performing a balanced excitation experiment, based on the experiment run by Song et al. (2000). Song et al. (2000) have shown that competitive Hebbian learning (Hebb, 1949) can be performed through STDP. The competition (induced by STDP) between the synapses can establish a bimodal distribution of the synaptic weights: either toward zero (*weak*) or the maximum (*strong*) values.

#### Using digital time window generator array

In this experiment, a single post-synaptic neuron is driven by 1024 TM synaptic adaptors, the TM addresses of which are from 0 × 0 to 0 × 3 FF. Their DA addresses are all the same: 0 × 0. That post-synaptic neuron has a single post-synaptic current generator that can generate both excitatory and inhibitory post-synaptic currents (EPSC and IPSC) modulated by the weights of the spikes arriving from different adaptors (Wang et al., 2014c). As the post-synaptic currents sum linearly in our model, only a PSC generator is needed in each neuron. Each adaptor was driven by an independent Poisson pre-synaptic spike train with the same average rate. We have tested the system with two firing rates: 10 and 20 Hz, whereas the firing rate of the post-synaptic neuron was 15 and 40 Hz respectively. The adaptors start with a uniform positive weight distribution. The size of the time window was fixed at 16 ms.

**Figure 10:**
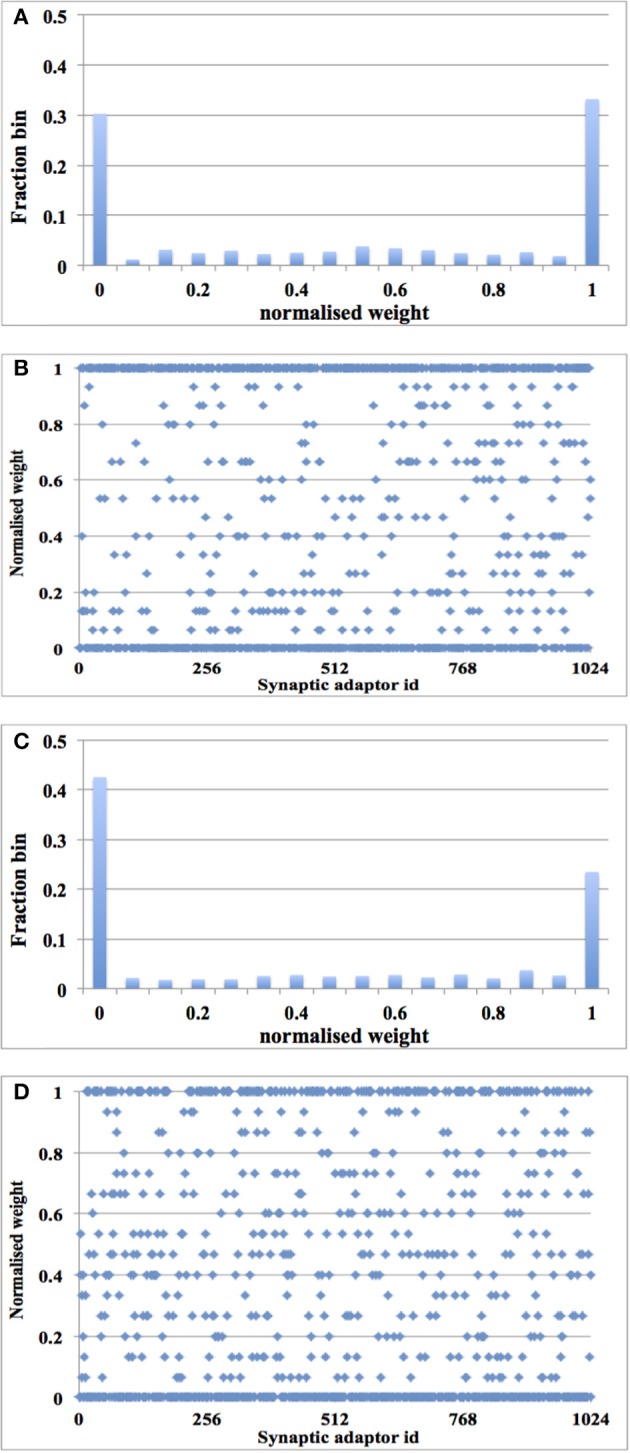
**Balanced excitation experiment with digital time window generator array**. **(A)** Weight distribution after 1.25 s of STDP for an input rate of 10 Hz. The bimodal distribution of *strong* and *weak* weights is apparent; **(B)** Scatter plot of the final weight distribution; **(C,D)** Same as **(A,B)**, but for an input rate of 20 Hz. Now more weights are *weak* than *strong*.

After 1.25 s of simulation, the distribution of synaptic weights converges to a steady-state condition with bimodal distribution of *strong* and *weak* weights (see Figure 10). Additionally, although our learning rule is considerably simplified when compared to that presented in Song et al. (2000), our system is capable of producing the same result: *for low input rates, more synaptic adaptors approach the upper limit, and for high input rates, more are pushed toward zero* (Song et al., 2000).

#### Using aVLSI time window generator array

**Figure 11:**
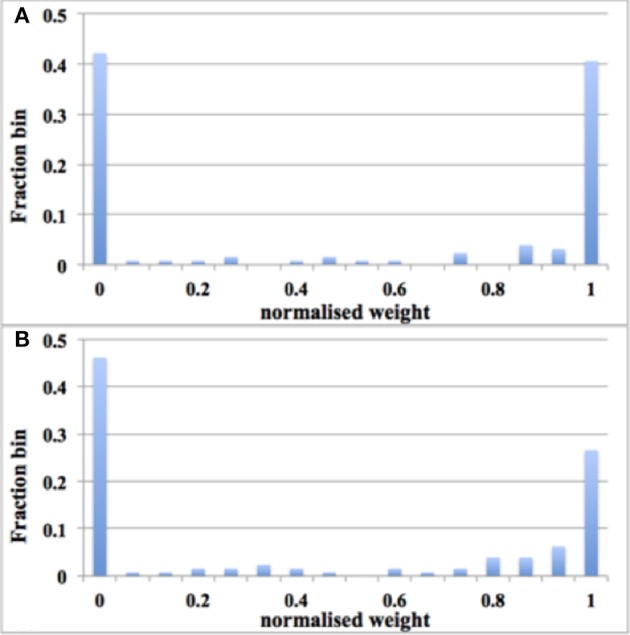
**Balanced excitation experiment with aVLSI time window generator array**. **(A)** Weight distribution after 1.25 s of STDP for an input rate of 10 Hz. The bimodal distribution of *strong* and *weak* weights is apparent; **(B)** Same as **(A)**, but for an input rate of 20 Hz. Now more weights are *weak* than *strong*.

We ran the experiment with 128 aVLSI time window generators (this is due to the fact that we have only 180 aVLSI time window generators and powers of two are preferable in digital design) with all the settings the same as with the digital time window generator. After 1.25 s of simulation, despite the adaptor using a fixed adaptation step (set to 1 here), the distribution of synaptic weights converges to a steady-state condition with a bimodal distribution of *strong* and *weak* weights (see Figure 11). It is also capable of producing the result: the higher the input rates, the more the synaptic weight will be pushed toward zero.

#### Validation of the dynamic-assignment scheme

The previous two experiments have shown that the balanced excitation experiment works for a system with 128 TM STDP adaptors. To validate the dynamic-assignment scheme, we conducted an experiment for 16 runs with an input rate of 20 Hz and 128 digital time window generators. For each run, these 128 TM STDP adaptors were assigned a DA address in the range from 0 × 0000 to 0 × 1 E00 with a step of 0 × 200. After each run, we read out the weights of these 128 adaptors (from the FPGA) and then started another run with the next DA address. In other words, we kept using the same 128 TM STDP adaptors for all the 16 runs by using the dynamic-assignment scheme. Note, this experiment is only a proof-of-concept and we can dynamically assign the TM adaptors for all those 8 k DA addresses (0 × 0 to 0 × 1 FFF) as long as the constraint of the active rate is not violated.

**Figure 12:**
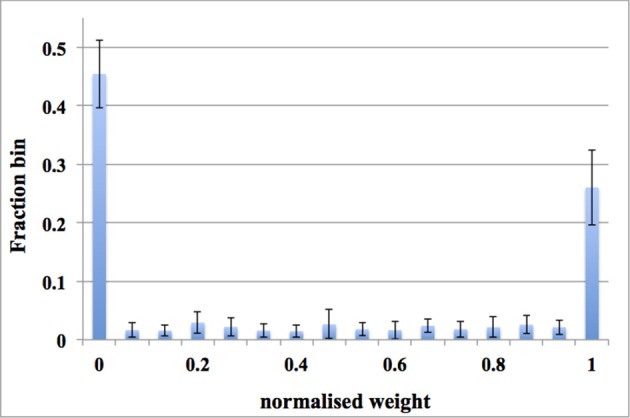
**Balanced excitation experiments using the dynamic-assignment scheme**. One TM STDP adaptor array (with 128 TM STDP adaptors) was dynamically assigned for 16 DA STDP adaptor arrays. The averaged weight distribution after 1.25 s of STDP for an input rate of 20 Hz. Note these data are averaged across all 16 runs. The bimodal distribution of *strong* and *weak* weights is apparent and more weights are *weak* than *strong*. Error bars are standard deviations of 16 runs.

For each run, the distribution of synaptic weights converges to a steady-state condition with a bimodal distribution of *strong* and *weak* weights. Figure 12 shows the average distribution of synaptic weights across all 16 runs. We first obtained the distribution of synaptic weights for each run and then averaged them. Since the input rate is 20 Hz, more synaptic weights were pushed toward zero, which matches the results presented in Figures 10C, 11B. For each run, the dynamic-assignment scheme has achieved a similar bimodal distribution of synaptic weights as the standard deviation of the results indicates. The dynamic-assignment scheme is therefore proved to be capable of performing what was designed to do: reusing hardware resources.

### Performance of STDDP

We have tested the performance of the dynamically-assigned STDDP adaptor array by performing a polychronization experiment. The term polychronization is used to indicate that several neurons can fire asynchronously but after traveling along axons with specific delays, their spikes will arrive at a post-synaptic neuron simultaneously, causing it to fire in turn (Izhikevich, 2006). Neural networks based on this principle are referred to as “polychronous” neural networks and are capable of storing and recalling quite complicated spatio-temporal patterns. In Wang et al. (2014d), we have concluded that the most important requirement of a hardware implementation of a polychronous network is to provide a strong time-locked relationship. This is indeed the motivation for us to develop the STDDP learning rule, which will fine-tune the axonal delays to the desired delay values.

#### Using digital time window generator array

In this experiment, we used 128 adaptors and a paired-pulse protocol: a single pair of pre- and post-synaptic spikes was sent to each of the adaptors periodically (every 32 time steps). During each period, each adaptor will receive one and only one pre-synaptic spike, the arrival time of which is randomized between time step 1 and 15. Additionally, during each period, each adaptor will receive one and only one post-synaptic spike, the arrival time is set to be time step 16. These spike pairs remain the same in each period. All the axonal delays are initialized to be zero. In each period, for each adaptor, a delay adaptation will be performed if the axonal delay has not been tuned to the desired delay. Hence, theoretically, after 15 times of STDDP, all the delayed pre-synaptic spikes from these 128 adaptors will fire simultaneously (each at its own time slot) at time step 16.

**Figure 13:**
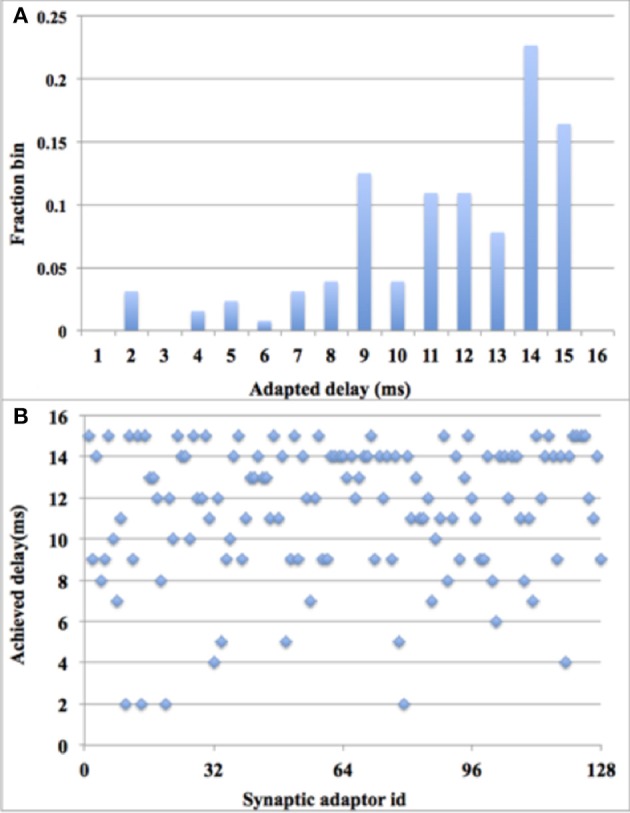
**Polychronization experiment with digital time window generator array**. **(A)** Delay distribution after 15 times of STDDP; **(B)**. Scatter plot of the final delay distribution.

This theoretical behavior was confirmed via measurements on the FPGA. Since plotting 128 delayed pre-synaptic spike that fire at the same time is meaningless, we chose instead to show the delay distribution after 15 times of STDDP and the scatter plot of the final delay distribution (see Figure 13). It might be noticed that the final delays are not uniformly distributed, which indicated that more pre-synaptic spikes arrive at the early part of that period than the ones arrive at the later part. The system has performed the polychronization experiment successfully since all the axonal delays have been fine-tuned to the desired values, which are the time differences between pre- and post-synaptic spikes.

#### Using aVLSI time window generator array

**Figure 14:**
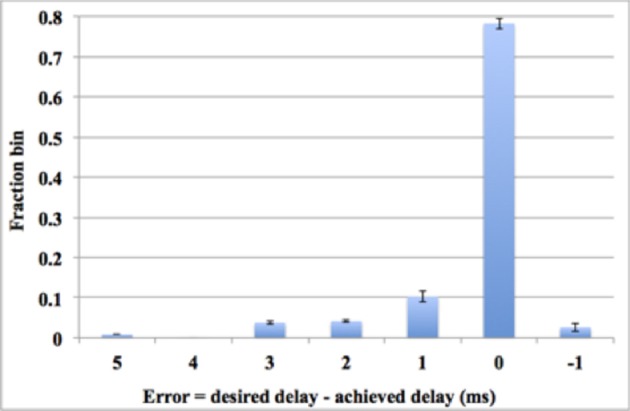
**Errors between the achieved delays and the desired values**. Error bars are standard deviations of 10 runs.

The digital time window generator can generate any given desired size of the time window (from 1 to 15 ms, in a time-step of 1 ms). But due to process variation and device mismatch, it is impossible to tune all the aVLSI time window generators with such accuracy. To compare the performance with its digital counterpart, we tested the system with all the settings the same as with the digital time window generator conducting 10 test runs for statistical purposes. Figure 14 shows the errors between the achieved delays and the desired delays. Note these data are averaged across all 10 runs. As the results showed that 78% of the achieved delays match the desired delays perfectly (within one time step). This number will go up to ~91% when we counted in the achieved delays with an error of ± 1 ms, both of which will still contribute to the process of the polychronization (Wang et al., 2013b). Thus, only a small fraction of the achieved delays (less than 9%), will be unable to contribute to the network. The standard deviation of the results indicates that the aVLSI time window generator array has achieved a fair variability (the averaged stand deviation is 0.006).

Compared to our previous work that implemented the same STDDP learning rule with fully aVLSI circuits (Wang et al., 2014d), this mixed-signal solution achieved a much better performance in terms of accuracy and density. More importantly, this mixed-signal solution stores the axonal delays in the digital memory, which is non-volatile and much more compact. The work reported here was developed with the lessons that we have learnt from our previous work that suffered a lot from the intrinsic difficulties of the aVLSI circuits, e.g., coupling noises, leakage currents, process variations, and device mismatch.

## Discussion

Since our goal aims for the maximum network size, our future work will focus on scaling up the network that we have presented here. For a system running at 266 MHz, we can achieve 256 k TM adaptors with one physical adaptor for a sub-millisecond time resolution. Given that we can implement 8 k DA STDP adaptors with a single TM adaptor, we can achieve 256 k × 8 k = 2G DA adaptors. With our bistable synaptic weight, which can be stored with a single bit, the total memory needed for this implementation is 2 Gb. It is clear that on-chip SRAM, which provides usually less than tens of megabits of storage, will not be able to meet this requirement. Among various external memory solutions, dynamic random access memory (DRAM) is the best candidate to provide the required storage because of its large storage capacity. High-end FPGA boards, such as Altera's DE5 board and Xilinx's VC709, usually contain two DDR3 SDRAM memories, each of which can currently support a maximum capacity of 64 Gb, and thus would allow us to implement 64G DA adaptors using only 64 physical adaptors. The corresponding TM adaptor arrays will need 64 × 8k × 4 bit = 2 Mb for the weight memory, which can easily be implemented using the on-chip SRAM. For the same system, the digital time window generator array would also need 2 Mb of storage.

In addition to the storage requirements, we also need to analyze the communications bandwidth requirement, which is generally the bottleneck for time-multiplexed implementations. The theoretically required bandwidth for 64 physical adaptors is 64 × 1 bit = 64 bits/clock cycle for both reading and writing. The DDR3 SDRAM is a single port device and the read/write operations cannot happen simultaneously. Thus, the required bandwidth of the SDRAM communication has to be doubled to 128 bits/clock cycle. Fortunately, the maximum theoretical bandwidth of one DDR3 SDRAM memory (when running at 1066 MHz) on an Altera DE5 board is 512 bits/clock cycle and even when considering that DDR3 memory typically only achieves 70% of that theoretical maximum bandwidth, there should be ample bandwidth to achieve the desired 128 bits/clock cycle. The reason for the reduced maximum bandwidth of the SDRAM is due to the need for flow control, which needs to take into consideration the bus turnaround time, memory refresh, finite burst length, and random access latency. All these will make the architecture of the system significantly more complex.

The cache structure was introduced to solve these difficulties. Firstly, it greatly reduces the bandwidth requirement to use external memory since the accessing (read/write) of the Master RAM will only be performed when needed and new values are not required to be available from memory every time slot. The reserved bandwidth can be used for other purposes, e.g., routing the spikes with look up tables. Secondly, this cache structure plus the fully-pipelined design style significantly ease the use of the off-chip memory. The pipeline of accessing the Master RAM can be simply reconfigured with different latency values to handle different flow control requirements.

The number of physical adaptors, i.e. the ones that can be activated simultaneously, will increase linearly with the number of available Slice LUTs, which are usually the bottleneck for high performance FPGA designs. But in our system, the design of the physical adaptor costs only a few LUTs and plenty of resources are left for additional physical adaptors or other systems components.

Based on the above calculations, we can conclude that it is practical to scale the proposed system up to a system with 64G DA adaptors on a commercial off-the-shelf high-end FPGA. The key to achieving this is to balance the number of physical adaptors (to achieve the best utilization of available hardware resources on the FPGA), the time-multiplexing rate (for a sub-millisecond time resolution), and the bandwidth and storage capacity of the memory.

Our aVLSI implementation is nowhere near as scalable as the digital implementation, since it can only be scaled up by implementing more physical copies of the aVLSI module. However, the introduction of the dynamic-assigning approach allows 8 k DA analog time window generators to be achieved with only a single physical time window generator. Above all, the motivation to develop the aVLSI implementation in the proposed system is for enhancement of the simulations.

### Conflict of interest statement

The authors declare that the research was conducted in the absence of any commercial or financial relationships that could be construed as a potential conflict of interest.
